# Photothermic Release of Curcumin for Antimicrobial Photodynamic Therapy

**DOI:** 10.1021/acsomega.5c06786

**Published:** 2025-11-20

**Authors:** Jeffersson K. Trigo-Gutierrez, Serena Medaglia, Elena Aznar, Ramón Martínez-Máñez, Ewerton G. O. Mima

**Affiliations:** † 291980 Universidad Privada Franz Tamayo, Facultad de Ciencias de la Salud, Carrera de Odontología, sede La Paz 1855, Bolivia; ‡ Department of Dental Materials and Prosthodontics, School of Dentistry, São Paulo State University (Unesp), Araraquara, São Paulo 14801-903, Brazil; § Instituto Interuniversitario de Investigación de Reconocimiento Molecular y Desarrollo Tecnológico (IDM), 16774 Universitat Politècnica de Valencia, Universitat de València, 46022 Valencia, Spain; ∥ Unidad Mixta de Investigación en Nanomedicina y Sensores, Universitat Politècnica de València, Instituto de Investigación Sanitaria La Fe (IISLAFE), 46026 Valencia, Spain; ⊥ CIBER de Bioingeniería, Biomateriales y Nanomedicina (CIBER-BBN), Instituto de Salud Carlos III, 28029 Madrid, Spain; # Departamento de Química, Universidad Politécnica de Valencia, 46022 Valencia, Spain; ∇ Unidad Mixta UPV-CIPF de Investigación en Mecanismos de Enfermedades y Nanomedicina, Universitat Politècnica de València, Centro de Investigación Príncipe Felipe, 46022 Valencia, Spain

## Abstract

Drug delivery systems (DDS) are promising tools to enhance antimicrobial Photodynamic Therapy (aPDT) by improving the targeted delivery and controlled release of photosensitizers. In this study, we introduce a light-responsive DDS based on curcumin-loaded mesoporous silica nanoparticles featuring a gold nanostar core and paraffin capping, designed specifically for near-infrared (NIR)-triggered photothermal release. This multicomponent nanoplatform uniquely combines photothermal activation with light-controlled drug delivery for antimicrobial applications. The synthesized nanoparticles exhibited a mean diameter below 500 nm, a polydispersity index of 0.154, and a surface charge of −21.9 mV. Upon NIR irradiation at 1200 J/cm^2^, curcumin release was approximately 90%. In planktonic bacterial cultures, aPDT mediated by this system led to reductions of 3.16 log_1__0_ and 2.18 log_1__0_ in colony-forming units (CFUs) for *Staphylococcus aureus* and *Pseudomonas aeruginosa*, respectively. For bacterial biofilms, a higher curcumin concentration (1000 μg/mL) resulted in CFU reductions of 2.16 log_1__0_ and 1.77 log_1__0_ for *S. aureus* and *P. aeruginosa*, respectively. This study demonstrated a NIR-activated nanocarrier for the controlled release of curcumin and effective inactivation of both planktonic and biofilm-associated bacteria—offering a new approach to improve the precision and efficacy of aPDT.

## Graphical abstract

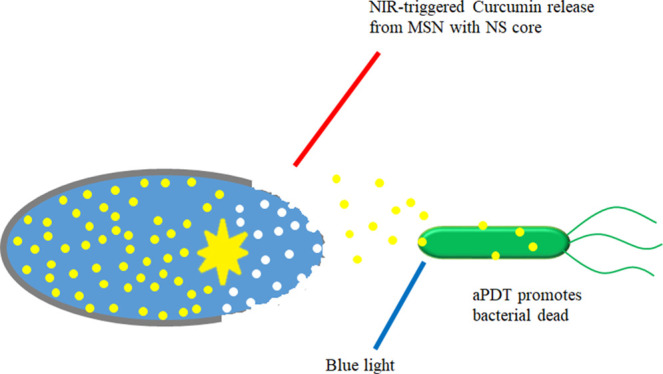

## Introduction

Bacteria have the ability to adhere to natural and artificial surfaces, forming structured communities known as biofilms in which cells are embedded in a self-produced extracellular polymeric matrix. This matrix protects the microorganisms from antimicrobial agents and the host’s innate immune system.^1^ Biofilm-specific features such as the presence of persister cells, impaired diffusion of antimicrobial agents, and increased tolerance have been associated with the failure of many conventional treatments.^2^

Among the most clinically relevant biofilm-forming pathogens is the Gram-positive bacterium *Staphylococcus aureus*, an opportunistic human pathogen responsible for pneumonia, various skin and soft tissue infections,^3^ and a major contributor to nosocomial infections.^4^ Similarly, the Gram-negative bacterium *Pseudomonas aeruginosa* is another important nosocomial pathogen, affecting approximately two million patients annually and contributing to an estimated 90,000 deaths each year.^5^

Given the global threat posed by antimicrobial resistance, there is an urgent need for alternative therapeutic strategies. One promising approach is antimicrobial Photodynamic Therapy (aPDT), which involves the activation of photosensitizers (PS) by light, leading to the generation of reactive oxygen species (ROS) that kill microorganisms.^6^ The great advantage of aPDT over conventional antimicrobials (antibiotics) is that the development of resistance is unlikely due to its nonselective and oxidative characteristics that target any component of the microbial cell, such as its cellular membrane, organelles, and DNA.^7,8^ Moreover, some recent investigations have demonstrated that aPDT can reverse the antimicrobial resistance, rendering the cells susceptible to the conventional antimicrobials.^9,10^

Among potential PS candidates, curcumin (CUR)—a natural compound extracted from the rhizomes of *Curcuma longa* L.—has attracted interest due to its anti-inflammatory, antioxidant, antimicrobial, and anticancer properties.^11^ CUR has demonstrated antimicrobial efficacy in aPDT against both planktonic and biofilm-forming bacteria and fungi.^12,13^ However, its hydrophobicity, low stability in aqueous media, and limited bioavailability hinder its clinical application.^14,15^

To overcome these challenges, drug delivery systems (DDS) have been employed to improve CUR’s stability, bioavailability, and targeting efficiency.^12–15^ Nanotechnology-based DDS, in particular, offer enhanced interaction with the complex biofilm structure and can be engineered to act at different stages of biofilm development.^16^ Several nanocarriers have been investigated for CUR delivery, including micelles, cyclodextrins, liposomes, nanoemulsions, polymeric nanoparticles, and metallic nanoparticles.^17^

Among these, mesoporous silica nanoparticles (MSNs) have emerged as a highly versatile platform due to their tunable surface chemistry, large surface area and pore volume, good biocompatibility, biodegradability, and ability to protect CUR from degradation.^18^ For example, CUR-loaded MSNs functionalized with polymyxin B have been applied in aPDT against planktonic and biofilm forms of *P. aeruginosa*, *Escherichia coli*, and *Staphylococcus epidermidis*. The system demonstrated effective bacterial eradication at low CUR concentrations (0.1–10 μg/mL) under blue light irradiation, with enhanced antimicrobial activity compared to free CUR.^19^

Despite these promising results, one of the limitations of current DDS is the slow and uncontrolled release of CUR, which can compromise aPDT efficacy.^12,13^ A key advantage of MSNs is their ability to be combined with molecular gates (also known as gatekeepers), which enable zero release under normal conditions and cargo release upon specific stimuli. Light-triggered systems, in particular, are attractive for PS delivery due to their ability to provide precise spatial and temporal control over drug release.^20^

For instance, photoresponsive micelles synthesized using *o*-nitrobenzyl and PEG have been used to deliver CUR against planktonic and biofilm forms of methicillin-resistant *S. aureus*, *P. aeruginosa*, and *Candida albicans*. Photocleavage of the micelle structure led to enhanced CUR release and increased antimicrobial efficacy compared to conventional micelles (e.g., Pluronic F127 or P123) and free CUR.^21^ Another class of light-responsive systems exploits the surface plasmon resonance (SPR) and photothermal effects of gold nanostructures under near-infrared (NIR) irradiation (700–950 nm). NIR light penetrates deeper into biological tissues and is converted into localized heat by gold nanoparticles, triggering drug release.^20^ For example, a hybrid nanosystem composed of mesoporous silica, copper, and silver was used to encapsulate CUR and irradiated with 72 J/cm^2^ of light to eradicate *E. coli* planktonic cultures.^22^ In another study, gold nanostars coated with mesoporous silica (AuNSt@mSiO_2_) and capped with paraffin (heneicosane) were used to photothermally release doxorubicin, demonstrating efficient control over drug release.^23^

Despite these advances, there are no previous reports combining MSN, gold nanostars (AuNSt), or CUR for aPDT applications. In this work, we report the synthesis of CUR-loaded MSN containing a gold nanostar core and capped with paraffin (AuNSt@mSiO_2_@CUR@paraffin). We evaluated this nanosystem in aPDT against planktonic and biofilm forms of *S. aureus* and *P. aeruginosa*, aiming to explore its potential for light-triggered antimicrobial therapy.

## Results and Discussion

### Synthesis of Nanocarrier

Gold nanoseeds (AuNsds) were synthesized via the reduction of gold chloride in the presence of sodium citrate, yielding particles with a hydrodynamic diameter of approximately 63.3 nm, as determined by dynamic light scattering (DLS) (Table 1). The nucleation and growth of gold nanoparticles can be influenced by the concentrations of gold chloride and sodium citrate since the final particle size depends on the number of nucleation sites over which the available gold is distributed.^24^ Another critical factor in AuNsd formation is the reaction temperature (100 °C), which, while accelerating the reduction process, also contributes to increased particle size.^25^ The Ultraviolet–visible (UV-vis) absorption spectrum of the synthesized AuNsds exhibited a characteristic surface plasmon resonance (SPR) band at 520 nm (Figure 1A), consistent with previously reported data.^26^

**Table 1:** Hydrodynamic Diameter, Polydispersity Index (PDI), and Zeta Potential Values for Each Step in the Synthesis of AuNSt@mSiO_2_@CUR@paraffin, Measured by Dynamic Light Scattering (DLS)^a^

	size (nm)	PDI (a.u.)	zeta potential (mV)
AuNsds	63.033 ± 9.41	0.149 ± 0.01	--
AuNSt	146.23 ± 18.94	0.256 ± 0.09	--
AuNst@mSiO_2_	243.83 ± 22.50	0.081 ± 0.02	36.2 ± 6.2
AuNst@mSiO_2_ CTAB extracted	324.5 ± 14.87	0.221 ± 0.10	–29.4 ± 2.
AuNst@mSiO_2_@CUR@paraffin	431.14 ± 38.03	0.154 ± 0.04	–21.9 ± 2.1

a AuNsds: Gold nanoseeds, AuNP: gold nanoparticle, AuNst: gold nanostar, mSiO_2_: mesoporous silica, CTAB: cetyltrimethylammonium bromide, CUR: curcumin, --: not performed.

**Figure 1:**
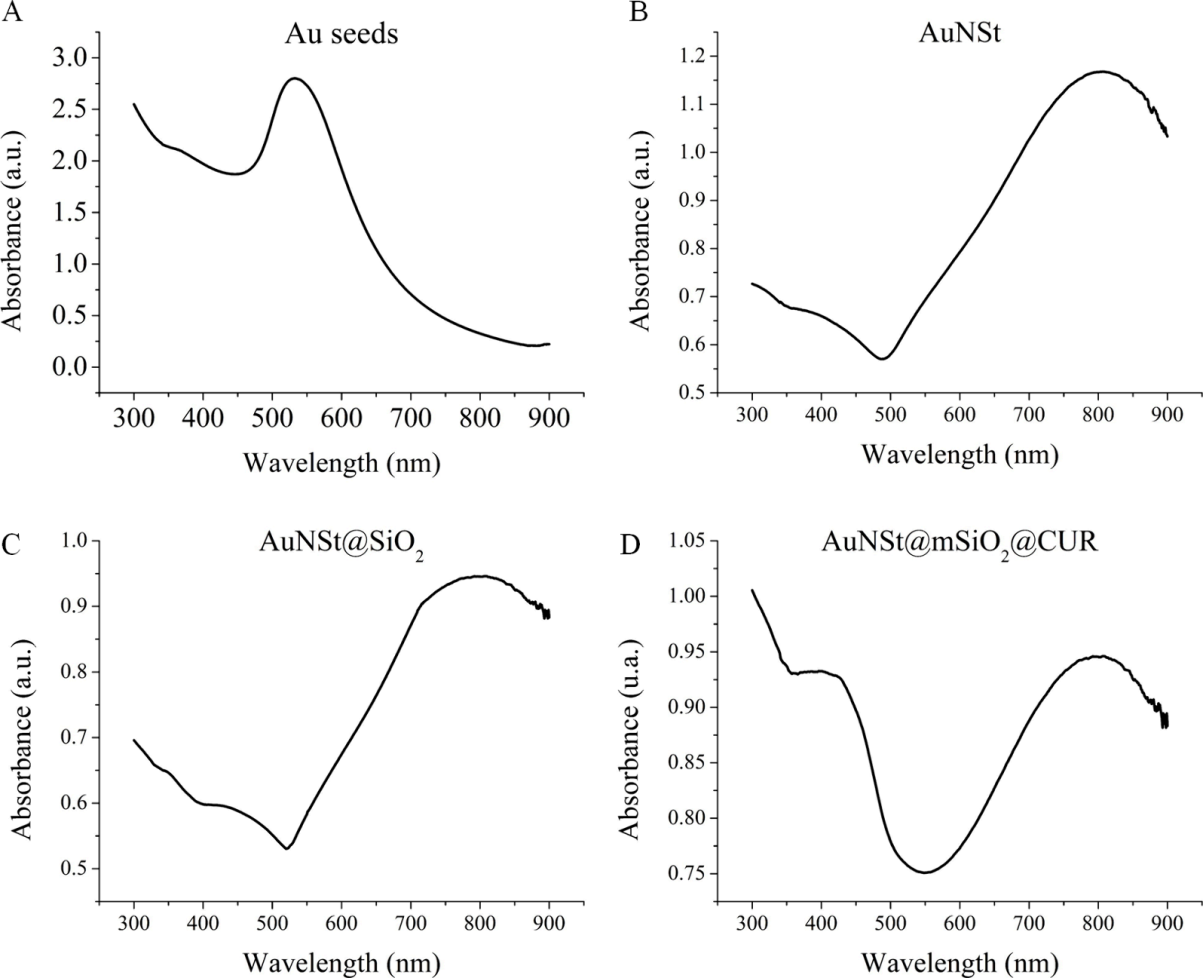
Ultraviolet–visible absorption spectra at different stages of nanocarrier synthesis: (A) Gold nanoseeds (AuNsds); (B) gold nanostars (AuNSts); (C) mesoporous silica-coated gold nanostars (AuNSt@mSiO_2_); (D) CUR-loaded mesoporous silica nanoparticles (AuNSt@mSiO_2_@CUR).

AuNsds were used as templates for the synthesis of gold nanostars (AuNSts). The reduction of Au^3^^+^ in the presence of polyvinylpyrrolidone (PVP) promoted the formation of star-shaped nanoparticles with multiple branches radiating from a central core, as observed in Figure 2A. The resulting AuNSts exhibited a hydrodynamic diameter of approximately 146 nm (Table 1). As reported in previous studies,^23,27^ the size and morphology of AuNSts can be modulated by varying the concentration of HAuCl_4_ and the size of the initial seed particles. The UV–vis absorption spectrum of AuNSts displayed a strong peak at 808 nm (Figure 1B), in agreement with the findings of Hernandez-Montoto et al.^23^ This red-shifted surface plasmon resonance (SPR) band is attributed to the increased number and sharpness of nanostar branches, which enhances light absorption in the NIR region.^28^

**Figure 2:**
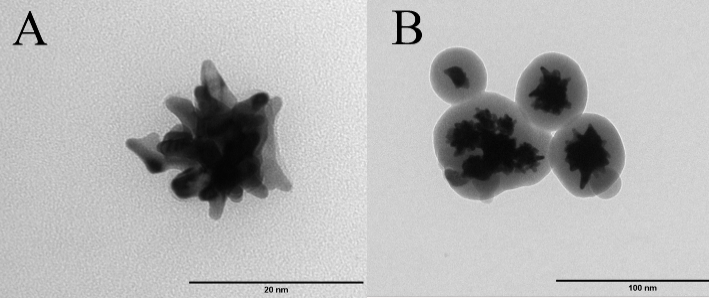
Transmission electron microscopy (TEM) images of nanocarrier components: (A) Gold nanostars (AuNSts) exhibiting branched morphology; (B) mesoporous silica-coated gold nanostars (AuNSt@mSiO_2_) showing a uniform silica shell around the core.

In the third step, MSNs were synthesized using a surfactant-templated sol–gel method, with cetyltrimethylammonium bromide (CTAB) micelles serving as pore-forming templates. The gold nanostars served as cores in the resulting hybrid nanostructures (AuNSt@mSiO_2_), as illustrated in Figure 2B. The AuNSt@mSiO_2_ particles showed a hydrodynamic diameter of approximately 243 nm and a zeta potential of +36 mV (Table 1), attributed to the positively charged CTAB surfactant on the particle surface.^23,29^ As shown in Figure 1C, the incorporation of the mesoporous silica shell did not significantly alter the optical absorption profile of the AuNSts.

Following the removal of the surfactant (CTAB) from AuNSt@mSiO_2_ nanoparticles, the zeta potential shifted to −29.4 mV (Table 1), which can be attributed to the presence of deprotonated silanol groups on the mesoporous silica surface.^29^ After extraction, the hydrodynamic size of the nanoparticles increased to 324 nm (Table 1).

Curcumin (CUR) was subsequently loaded into the mesoporous silica structure via a simple stirring method.^19^ The CUR-loaded nanoparticles were then functionalized with octadecyltrimethoxysilane and capped with heneicosane, resulting in the final nanocarrier formulation: AuNst@mSiO_2_@CUR@paraffin. This surface modification led to an increase in hydrodynamic diameter to 431 nm, while maintaining good monodispersity, with a polydispersity index (PDI) of 0.154 au.^30^ The zeta potential after CUR encapsulation and paraffin capping decreased to −21.9 mV (Table 1), indicating surface modification. A similar trend was reported by Ribeiro et al.,^31^ who observed a zeta potential shift from −20.7 to −16.7 mV.

The successful encapsulation of CUR was confirmed by UV–vis spectroscopy (Figure 1D), which displayed two characteristic absorption peaks: one at 808 nm corresponding to the AuNSt core, and another at 425 nm attributed to CUR. The entrapment efficiency (EE%) of CUR was calculated to be approximately 90%, in agreement with previous studies that reported an EE% of 87% for CUR-loaded MSN.^31^ A schematic overview of the synthesis steps and structural evolution of the nanocarrier is presented in Figure 3.

**Figure 3:**
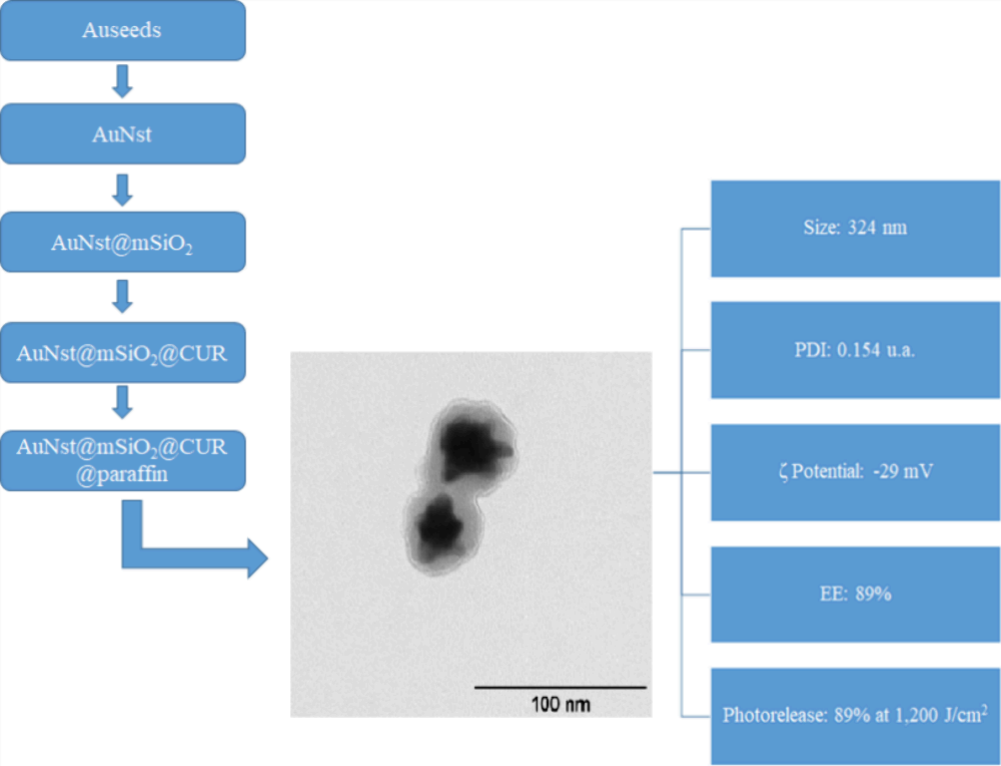
Schematic representation of the synthesis and functional performance of the AuNSt@mSiO_2_@CUR@paraffin nanocarrier, illustrating gold nanostar formation, mesoporous silica coating, curcumin loading, paraffin capping, and subsequent light-triggered release for antimicrobial Photodynamic Therapy (aPDT).

The photothermal release of curcumin (CUR) from AuNSt@mSiO_2_@CUR@paraffin nanoparticles was evaluated both in the absence and presence of NIR laser irradiation at 808 nm (1200 J/cm^2^ for 20 min). As shown in Figure 4, CUR release was negligible in the absence of light, indicating effective paraffin gating under ambient conditions. However, upon NIR irradiation, a significant increase in CUR release was observed, reaching approximately 90% after 20 min. This light-triggered release is attributed to the photothermal effect of the AuNSt core, which converts NIR light into localized heat, causing the melting of the paraffin gate and enabling controlled diffusion of CUR into the surrounding medium.^32^

**Figure 4:**
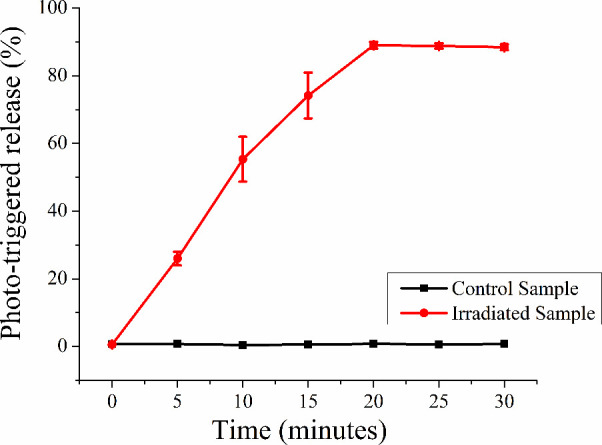
Photothermal release profile of curcumin (CUR) from AuNSt@mSiO_2_@CUR@paraffin nanoparticles. CUR release was monitored over 30 min in the absence (control) and presence of 808 nm laser irradiation (1200 J/cm^2^), demonstrating near-infrared-triggered release due to paraffin melting induced by the photothermal effect of the gold nanostar core.

MSNs are widely used for photosensitizer delivery due to their optical transparency, high surface area, and excellent biocompatibility.^33,34^ To evaluate the performance of the synthesized AuNSt@mSiO_2_@CUR@paraffin system in aPDT, different nanoparticle concentrations (5, 50, and 500 μg/mL) were tested against planktonic cultures of *S. aureus* and *P. aeruginosa*. The bacterial suspensions were incubated with the nanocarrier for 5 min (pre-irradiation time, PIT), followed by 20 min of 808 nm NIR laser irradiation (1200 J/cm^2^). Although a laser fluence of 1200 J/cm^2^ may be relatively high for biological applications, we maintained the same laser parameters used in the Release Assay—20 min of irradiation at an intensity of 1 W/cm^2^—in order to maximize CUR release, which reached approximately 90%. Lower fluences may not be sufficient to melt the paraffin gates and achieve complete CUR release, as observed in Figure 4. Given that fluence (J/cm^2^) is calculated as the product of light intensity (W/cm^2^) and irradiation time (s), the resulting fluence was indeed 1200 J/cm^2^. Subsequently, blue LED light (∼450 nm, power intensity 30 W/cm^2^) was applied at 36 J/cm^2^ (20 min)^12,21^ to activate CUR and generate ROS. The light fluence used for aPDT (36 J/cm^2^) was selected based on our previous studies,^12,21^ thus different energy densities were not evaluated. The experimental setup and control groups are summarized in Figure S1.

Regarding NIR irradiation, the effects of light on bacterial growth—whether stimulatory or inhibitory—depend on multiple factors, including the light intensity (fluence), wavelength, and specific bacterial species involved. Red and NIR light is known to induce cell proliferation primarily by stimulating the mitochondrial respiratory chain, enhancing ATP synthesis, and activating intracellular signaling cascades. This phenomenon, known as photobiomodulation, is well characterized in mammalian and host cells.^35,36^ In contrast, most studies of light irradiation on bacteria report inhibitory effects.^37^ However, some evidence suggests species-specific responses; for example, Nussbaum et al.^38^ reported that 810 nm low-level laser irradiation reduced the growth of *Pseudomonas aeruginosa* but increased the growth of *Escherichia coli*, highlighting the importance of both irradiance and bacterial type. The effect of light on bacteria is explained by the production of ROS, which in high amount are toxic to bacterial cells, but in low amount induce cell proliferation.^36,39^ However, a threshold level of ROS that distinguishes between stimulatory or inhibitory effects on bacterial growth has not been clearly established. In our study, the results obtained with planktonic cultures (Figure 5) showed no statistically significant difference in bacterial growth between the laser-irradiated group (Laser+) and the untreated control. This suggests that the laser fluence used in our experiments did not have a stimulatory or inhibitory effect on the bacterial proliferation under the conditions tested.

**Figure 5:**
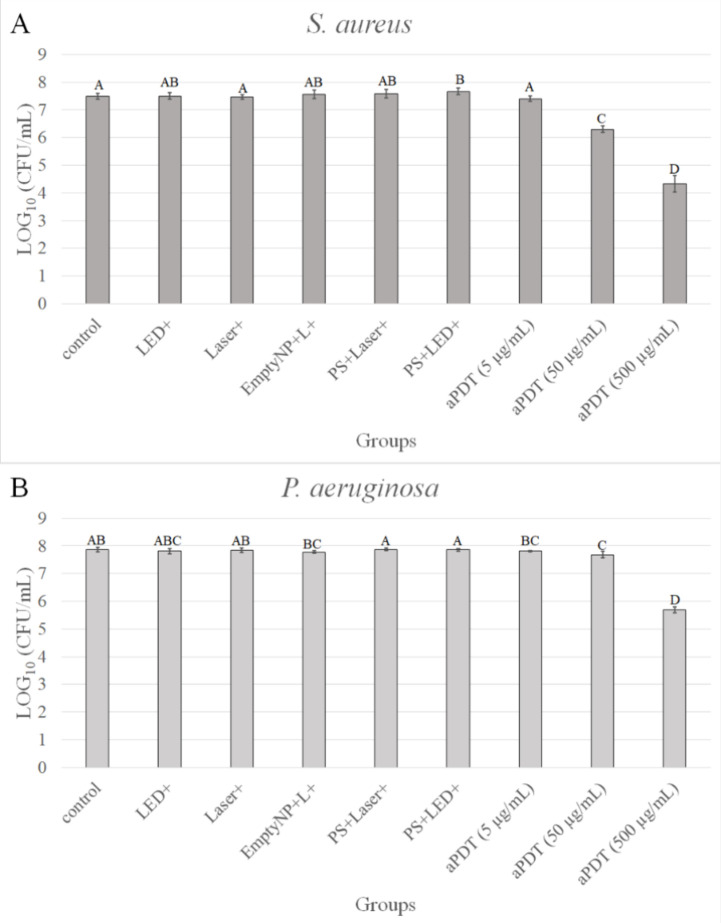
Antimicrobial photodynamic inactivation of planktonic cultures of *Staphylococcus aureus* (A) and *Pseudomonas aeruginosa* (B) treated with AuNSt@mSiO_2_@CUR@paraffin (photosensitizer, PS) at 5, 50, and 500 μg/mL. Bacterial suspensions were incubated with the PS for 20 min in the dark (pre-irradiation time), followed by near-infrared (NIR) laser irradiation (808 nm, 1200 J/cm^2^) to trigger curcumin (CUR) release. Blue LED irradiation (∼450 nm, 36 J/cm^2^) was subsequently applied to activate the CUR for antimicrobial Photodynamic Therapy (aPDT). Error bars represent standard deviation. Different letters indicate statistically significant differences among groups (ANOVA/Welch followed by Games–Howell post hoc test, *n* = 12). Experimental groups are further detailed in Figure S1 of Supporting Information.

The results of aPDT against planktonic bacteria are presented in Figure 5. For the Gram-positive *S. aureus*, treatment with AuNSt@mSiO_2_@CUR@paraffin at 50 and 500 μg/mL resulted in statistically significant reductions in bacterial viability compared to all other groups (*p* ≤ 0.001), with decreases of 1.19 and 3.16 log_1__0_(CFU/mL), respectively, relative to the untreated control (Figure 5A). In the case of Gram-negative *P. aeruginosa*, the 50 μg/mL dose led to a modest but significant reduction of 0.18 log_1__0_(CFU/mL) (*p* ≤ 0.029), while treatment with 500 μg/mL resulted in a substantial decrease of 2.18 log_1__0_(CFU/mL), with statistical significance versus all other groups (*p* ≤ 0.001) (Figure 5B).

Comparable findings were reported by Medaglia et al.,^19^ who observed up to a 5-log reduction in CFU counts for *S. epidermidis*, *E. coli*, and *P. aeruginosa* using CUR-loaded MSN functionalized with Polymyxin B. However, their nanocarrier system also exhibited antimicrobial effects in the absence of light possibly due to the intrinsic activity of the antibiotic.

CUR does possess intrinsic antimicrobial properties, but typically at higher concentrations.^40^ In our previous studies,^12,21^ we combined curcumin with blue light and observed that this combination enhanced CUR’s antimicrobial activity. We used a low, nontoxic concentration of CUR, as required in aPDT, where the PS must be nontoxic on its own. Otherwise, the observed antimicrobial effect cannot be attributed to a photodynamic mechanism.

Following the evaluation of aPDT against planktonic cultures, *S. aureus* and *P. aeruginosa* were cultivated as single-species biofilms for 48 h. aPDT was initially performed using AuNSt@mSiO_2_@CUR@paraffin at 500 μg/mL—the concentration that demonstrated the most pronounced antibacterial effect in planktonic assays. However, under these conditions, no significant reduction in the viable cell count was observed (data not shown). This outcome is likely due to the protective nature of the biofilm’s extracellular polymeric matrix, which limits the diffusion and penetration of PS.^41^

To overcome this barrier, the concentration of AuNSt@mSiO_2_@CUR@paraffin was increased to 1000 μg/mL, while maintaining the same preirradiation time (PIT), NIR laser (808 nm, 1200 J/cm^2^), and blue light (450 nm, 36 J/cm^2^) irradiation parameters used in the planktonic assay. Under these optimized conditions, aPDT treatment led to a significant reduction in *S. aureus* biofilm viability, with a decrease of 2.16 log_1__0_(CFU/mL) compared to the untreated control group (*p* ≤ 0.001) (Figure 6A). For *P. aeruginosa*, a similar trend was observed, with a reduction of 1.77 log_1__0_(CFU/mL) compared to the control (*p* ≤ 0.001) (Figure 6B).

**Figure 6:**
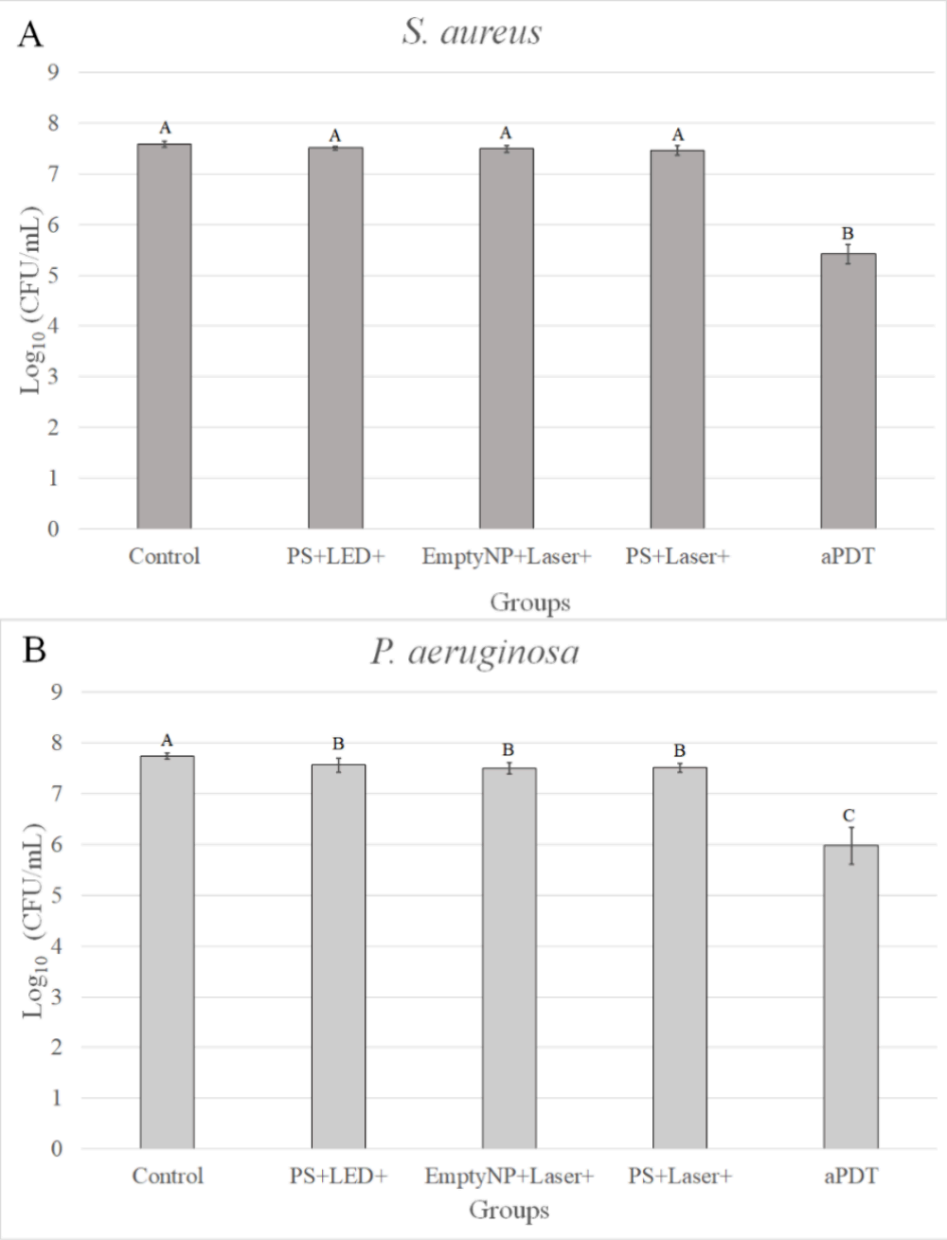
Mean log_1__0_(CFU/mL) values of single-species biofilms of *Staphylococcus aureus* (A) and *Pseudomonas aeruginosa* (B) treated with antimicrobial Photodynamic Therapy (aPDT) mediated by AuNSt@mSiO_2_@CUR@paraffin (PS) at 1000 μg/mL. Biofilms were incubated with the photosensitizer (PS) for 20 min in the dark (pre-irradiation time), followed by near-infrared (NIR) laser irradiation (808 nm, 1200 J/cm^2^) to trigger curcumin (CUR) release, and then blue LED irradiation (∼450 nm, 36 J/cm^2^) to activate photodynamic action. Error bars represent standard deviation. Different letters indicate statistically significant differences among groups (ANOVA/Welch with Games–Howell post hoc test, *n* = 8). Experimental groups: Control: untreated bacterial biofilms; PS+LED+: samples incubated with the PS and irradiated by LED; EmptyNP+L+: samples incubated with empty nanoparticles (nanocarrier without CUR) following laser irradiation; PS+Laser+: samples incubated with the PS and irradiated by laser; aPDT: samples incubated with the PS followed by laser and LED irradiation.

This study presents a preliminary study focused on the synthesis of a nanosystem and its evaluation as a PS for use in aPDT, with the goal of improving the solubility of CUR to facilitate its potential clinical application. At this stage, we prioritized thorough characterization of the synthesized nanosystem. Consequently, we did not undertake a more extensive evaluation of its antimicrobial activity, such as testing against other microbial kingdoms (e.g., fungi), assessing polymicrobial biofilms, evaluating cytotoxicity against host cells and tissues, or conducting animal infection models. Moreover, using two wavelengths (infrared and visible blue) may increase the cost of the process. However, some clinical settings already use blue light, for instance, for the photopolymerization of composite resins in a dental office, which can be also used for aPDT, and, in this case, only an infrared light would be needed to be added to use the nanosystem.

Our results demonstrated that a higher concentration of PS was required to achieve a significant reduction in biofilm viability compared to planktonic cells, reinforcing the well-known challenge that biofilms pose in antimicrobial treatment. Future studies should focus on combining the nanosystem with agents capable of disrupting or modifying the extracellular polymeric matrix as well as evaluating its application in more complex models—such as multispecies biofilms, tissues containing microorganisms or biofilms, and in vivo infection models. Furthermore, given that CUR exhibits other therapeutic properties, the nanosystem may also be explored as an anti-inflammatory, antioxidant, or anticancer agent; however, these applications require specifically designed studies.

## Conclusions

In summary, the smart nanoplatform AuNSt@mSiO_2_@CUR@paraffin was successfully synthesized and characterized, demonstrating an efficient NIR-triggered release of curcumin. This validates its functionality as a light-responsive DDS. The nanocarrier exhibited significant antimicrobial photodynamic activity against both *S. aureus* and *P. aeruginosa*, effectively reducing bacterial viability in both planktonic cultures and more resilient single-species biofilms.

These findings underscore the potential of this nanostructured system to address critical challenges in antimicrobial therapy, particularly in the treatment of biofilm-associated infections, which are often tolerant to conventional antibiotics. Moving forward, further investigation is warranted in more complex biological systems, including multispecies biofilms that better mimic clinical conditions as well as in vivo animal models to assess safety, pharmacokinetics, and therapeutic efficacy under physiologically relevant environments. Such studies will be essential to advance the clinical translation of AuNSt@mSiO_2_@CUR@paraffin-mediated aPDT as a promising adjunct or alternative approach in combating persistent bacterial infections.

## Methods

### General

Polivinil pirrolidone (PVP-MW 10000), citrate, gold auric-III-(HAuCl_4_), sodium citrate 1%, CTAB, *N,N-*dimethylformamide (DMF), curcumin (CUR), tetraethoxysilane (TEOS), acetonitrile (ACN), and octadecyltrimethoxysilane (OCT) were purchased from Sigma-Aldrich Qumica S.L., Madrid, Spain. Equipment used in this study included a UV–visible spectrophotometer (JASCO V-650 UV/vis, Easton, USA), a fluorimeter (JASCO, FP-8300, Hitachi High Technologies, Minato-ku, Tokyo, Japan), a centrifuge (Eppendorf, 5702, Fisher Scientific S.L, Alcobendas (Madrid), Spain), a laser NIR (developed by the Engineering Department of Universitat Politècnica de València), and a Blue LED light at ∼450 nm with a power of 30 mW/cm^2^ (Bulbs Lighting Co. Shenzhen, China).

### Synthesis of Gold Nanoseeds

Gold nanoseeds (AuNsds) were synthesized following a previously described protocol with slight modifications.^24,25^ Briefly, a double-necked round-bottomed flask containing 100 mL of distilled water and 70 μL of HAuCl_4_ (1.444 M) was heated to 100 °C under reflux. Once boiling, 15 mL of a 1% (w/v) aqueous sodium citrate solution was added rapidly under magnetic stirring (1200 rpm), and the mixture was maintained at 100 °C for 15 min. Afterward, the reaction was allowed to cool and stand at room temperature for 24 h.

To functionalize the AuNsds with PVP, 20 mL of the resulting AuNsds suspension was mixed with 1 mL of an aqueous PVP solution (500 mg/mL) in a clean double-neck round-bottom flask. The mixture was stirred magnetically at 1200 rpm for 18 h at room temperature. The AuNsds were then collected by centrifugation and resuspended in 1.5 mL of ethanol.^23^

### Synthesis of Gold Nanostars (AuNSt)

Gold nanostars (AuNSt) were synthesized using a seeded growth method as previously described.^23^ Briefly, 150 μL of an aqueous HAuCl_4_ solution (166 mM) was added dropwise into 45 mL of a PVP solution (30 mM) prepared in DMF. After stirring for 5 min, Au nanoseeds (AuNsds) at 5 mM were introduced into the mixture, which was then left undisturbed at 25 °C for 24 h to allow nanostar growth. The resulting AuNSt were collected by centrifugation at 9500 rpm for 20 min, washed once with distilled water, and resuspended for further use.^23^

### Synthesis of MSNs with Gold Nanostars (AuNst@mSiO_2_)

MSNs were synthesized by using a surfactant-templated method. In a double-neck round-bottom flask, 20 mL of ethanol was added to 50 mL of an aqueous solution of CTAB (6.6 mM). The solution was purged with argon (Ar) gas for 1 h to create an inert atmosphere. Then, 50 μL of aqueous ammonia solution (32%) and 5 mM AuNSt suspension were introduced into the flask. After 5 min, 40 μL of tetraethoxysilane (TEOS) was added dropwise to initiate silica condensation. The reaction was maintained under an Ar atmosphere and stirred for 24 h to complete the formation of AuNSt@mSiO_2_ nanoparticles.^23^

### Synthesis of AuNSt@mSiO2@CUR@paraffin

A stock solution of curcumin (CUR) was prepared by dissolving CUR in acetonitrile at a concentration of 1 mg/mL. This solution was added to a vial containing AuNSt@mSiO_2_ nanoparticles at 1 mg/mL. The mixture was stirred magnetically at 400 rpm for 1 h at 25 °C to allow CUR loading. Subsequently, 20 μL of octadecyltrimethoxysilane (OCT) was added, and the stirring continued at 25 °C for an additional 12 h to facilitate surface functionalization.

Afterward, the nanoparticles were centrifuged and washed with 0.5% aqueous ACN to remove unbound CUR. The absorbance of the supernatant was measured spectrophotometrically, and the amount of CUR loaded was calculated using a calibration curve (Figure S2). Finally, the CUR-loaded nanoparticles were vacuum-dried for further use.^42^

The entrapment efficiency was calculated using the following formula:

$$\mathrm{EE}\%=\frac{\mathrm{Encapsulated}\;\mathrm{amount}}{\mathrm{Total}\;\mathrm{amount}\;\mathrm{of}\;\mathrm{CUR}}\times 100$$

For the incorporation of the molecular gate, CUR-loaded nanoparticles were diluted in 10 mL of *n*-hexane. Subsequently, 1 mL of a heneicosane solution (20 mg/mL in *n*-hexane) was added to the dispersion. The mixture was sonicated for 1 min and then homogenized using a vortex mixer for 20 s. This sonication and vortexing cycle was repeated continuously for 30 min to ensure uniform coating of the nanoparticles with the paraffin gate.^23^

### Nanoparticle Characterization

The AuNsds, AuNSt, AuNst@mSiO_2_, AuNSt@mSiO2@CUR, and AuNSt@mSiO_2_@CUR@paraffin were characterized as follows: Absorption spectra of nanoparticles were recorded using a UV–visible spectrophotometer over the range of 300 to 900 nm. Nanoparticle samples were diluted at 1 mg/mL and placed in cuvettes, and their absorbance was measured spectrophotometrically. DLS was used to determine the hydrodynamic size and polydispersity index (PDI) of the nanoparticles, while zeta potential measurements assessed their surface charge. For AuNst@mSiO_2_, zeta potential was measured at different stages: before and after CTAB extraction, after CUR loading, and following paraffin capping. Transmission electron microscopy (TEM) was employed to analyze the nanoparticle size and morphology. Samples at 1 mg/mL were deposited onto silica grids and air-dried at room temperature prior to imaging.

Light-triggered release studies were performed using an 808 nm NIR laser (1 W/cm^2^). The laser device was set 1 cm above the 96-well flat-bottom microplate^23^ with 90° angle of irradiation at 25 °C. Samples were irradiated at various time points (0, 5, 10, 15, 20, 25, and 30 min). After each irradiation interval, samples were centrifuged at 16,670 × g for 5 min at 5 °C, and the fluorescence of the supernatant was measured at 498 nm. Control samples were kept in the dark for comparison.

### aPDT against Planktonic Cultures

Colonies of *Staphylococcus aureus* (CECT 240), grown on Müller Hinton Agar (MHA), and *Pseudomonas aeruginosa* (ATCC 15442), grown on Pseudomonas Agar (PA), were transferred to Müeller-Hiton Broth (for *S. aureus*) and Luria-Bertoni Broth (for *P. aeruginosa*). The cultures were standardized at 4.72 × 10^7^(±3.9 × 10^6^) Colony Forming Units per milliliter (CFU/mL) for *S. aureus* and 6.97 × 10^7^(±7.52 × 10^6^) CFU/mL for *P. aeruginosa*. A volume of 100 μL of each microbial suspension was transferred to individual wells of a 96-well flat-bottom microplate. Subsequently, 100 μL of AuNSt@mSiO_2_@CUR@paraffin nanoparticles (1 mg/mL in aqueous solution) were added to each well, resulting in a final nanoparticle concentration of 500 μg/mL. Additional wells were prepared with final concentrations of 50 and 5 μg/mL to assess concentration-dependent effects. The plates were incubated in the dark by 20 min (pre-irradiation time, PIT) at 25 °C, followed by laser irradiation at 808 nm (20 min, 1200 J/cm^2^) to trigger the release of CUR as described above for the release assay. Immediately after, a blue LED light (∼450 nm, 36 J/cm^2^) placed 20 cm perpendicular (90°) of culture microplate^19^ was used to irradiate the samples for 20 min to initiate photodynamic microbial inactivation (aPDT).

Control groups were included to assess the effects of light and nanoparticles individually: PS+LED+: Samples treated with the nanoparticle but only irradiated with blue light; PS+Laser+: Samples treated with the nanoparticle but only irradiated with an NIR laser; EmptyNP+L+: Samples treated with empty nanoparticles (without CUR) and irradiated with the laser; LED+ and Laser+: Microbial suspensions in PBS (no nanoparticles or PS) irradiated with blue light or NIR laser, respectively; Control: Untreated bacterial suspensions (no irradiation or nanoparticle).

Following treatment, 100 μL of each bacterial suspension was transferred to microtubes containing 900 μL of PBS and vortexed for 1 min. Serial 10-fold dilutions were prepared, and 25 μL aliquots of each dilution were plated in duplicate onto MHA (for *S. aureus*) and PA (for *P. aeruginosa*). The plates were incubated at 37 °C for 48 h. After incubation, colonies were counted, and the microbial viability was calculated by using the following formula:

CFU/mL=meanofcoloniesinduplicate×10^dilution0.025mL

### aPDT against Single-Species Biofilms

To establish single-species biofilms of *S. aureus* and *P. aeruginosa*, 200 μL aliquots of each standardized bacterial suspension were transferred into individual wells of a flat-bottomed 96-well culture plate. The plates were incubated at 37 °C for 90 min to allow cell adhesion. After this adhesion phase, the wells were gently washed twice with 200 μL of sterile PBS to remove nonadherent cells. Then, 200 μL of the appropriate broth medium was added to each well, and the plates were incubated for 48 h at 37 °C for biofilm maturation. Bacterial biofilms are typically grown for 24 to 72 h, which is the time required for the production of extracellular matrix and for the biofilm to reach maturity.^43^ Our previous study^21^ also used 48 h biofilms of *S. aureus* and *P. aeruginosa*, and confocal images showed thick biofilms and CUR uptake. After 24 h, 100 μL of the medium was carefully removed from each well and replaced with 100 μL of fresh broth to maintain nutrient levels.

To evaluate the antimicrobial photodynamic effect of AuNSt@mSiO_2_@CUR@paraffin, only conditions that demonstrated activity in the planktonic assays were tested. Initially, biofilms were treated with 500 μg/mL nanoparticle formulation, but no reduction in viable cells was observed (data not shown). Therefore, a higher concentration (1 mg/mL) was evaluated. The culture medium was removed, and the wells were gently washed twice with PBS. The nanoparticle suspension was added and incubated under the same conditions described for planktonic assays (pre-irradiation in the dark for 20 min). Additional samples were incubated with AuNSt@mSiO_2_@CUR@paraffin that were irradiated with NIR laser or blue LED light only (PS+Laser+, PS+LED+ groups, respectively). Another group received empty nanoparticles following laser irradiation (EmptyNP+Laser+ group). The control group was incubated only with PBS. The following experimental groups were included: aPDT: Biofilms treated with AuNSt@mSiO_2_@CUR@paraffin (1 mg/mL), followed by NIR laser (808 nm, 1,200 J/cm^2^) and blue LED light (∼450 nm, 36 J/cm^2^) irradiation; PS+Laser+: Treated with the PS and irradiated with NIR laser only; PS+LED+: Treated with the PS and irradiated with blue LED light only; EmptyNP+Laser+: Treated with empty nanoparticles (without CUR) and irradiated with the NIR laser; Control: Untreated biofilms incubated with PBS. After treatment, the biofilms were mechanically disrupted using a pipet tip, serially diluted in PBS, and plated onto selective agar (MHA for *S. aureus*, PA for *P. aeruginosa*) for CFU/mL quantification, as described in the planktonic assay section.

### Statistical Analyses

Microbial assays with planktonic cultures were conducted in quadruplicate on three independent occasions (*n* = 12), while biofilm experiments were performed in quadruplicate on two separate occasions (*n* = 8). Data were assessed for normality and homogeneity of variances prior to analysis. Statistical comparisons were performed using ANOVA or Welch’s ANOVA, followed by Tukey’s or Games–Howell post-hoc tests, as appropriate. A significance level of 5% was adopted for all of the analyses.

## Supplementary Material



## Notes

Experimental conditions for aPDT against bacterial planktonic cultures and biofilms; and curcumin absorption spectrum and curcumin standard curve calibration (PDF)
