# Supplementary material for: Photothermic Release of Curcumin for Antimicrobial Photodynamic Therapy
Source: ACS Omega. 2025 Nov 20;10(48):58600–9. doi: 10.1021/acsomega.5c06786 (PMC12771119; doi:10.1021/acsomega.5c06786)
Supplement: Supplementary file 1 [file ao5c06786_si_001.pdf]

Photothermic release of curcumin for antimicrobial Photodynamic Therapy

Jeffersson K. Trigo-Gutierrez[1,2,3], *Serena Medaglia*[3,4,5], *Elena Aznar* [3,4,5,6,7], *Ramón Martínez-Máñez* [3,4,5,6,7], *Ewerton G. O. Mima*[2] *

[1]Universidad Privada Franz Tamayo, Facultad de Ciencias de la Salud, Carrera de Odontología, sede La Paz 1855, Bolivia

[2]Department of Dental Materials and Prosthodontics, School of Dentistry, São Paulo State University (Unesp), Araraquara, São Paulo 14801-903, Brazil.

[3] Instituto Interuniversitario de Investigación de Reconocimiento Molecular y Desarrollo Tecnológico (IDM), Universitat Politècnica de Valencia, Universitat de València, Spain

[4] Unidad Mixta de Investigación en Nanomedicina y Sensores, Universitat Politècnica de València, Instituto de Investigación Sanitaria La Fe (IISLAFE), 46026 Valencia, Spain

[5]CIBER de Bioingeniería, Biomateriales y Nanomedicina (CIBER-BBN), Instituto de Salud Carlos III, 28029 Madrid, Spain

[6]Departamento de Química, Universidad Politécnica de Valencia, 46022 Valencia, Spain

[7]Unidad Mixta UPV-CIPF de Investigación en Mecanismos de Enfermedades y Nanomedicina, Universitat Politècnica de València, Centro de Investigación Príncipe Felipe, Valencia, Spain

Supporting information

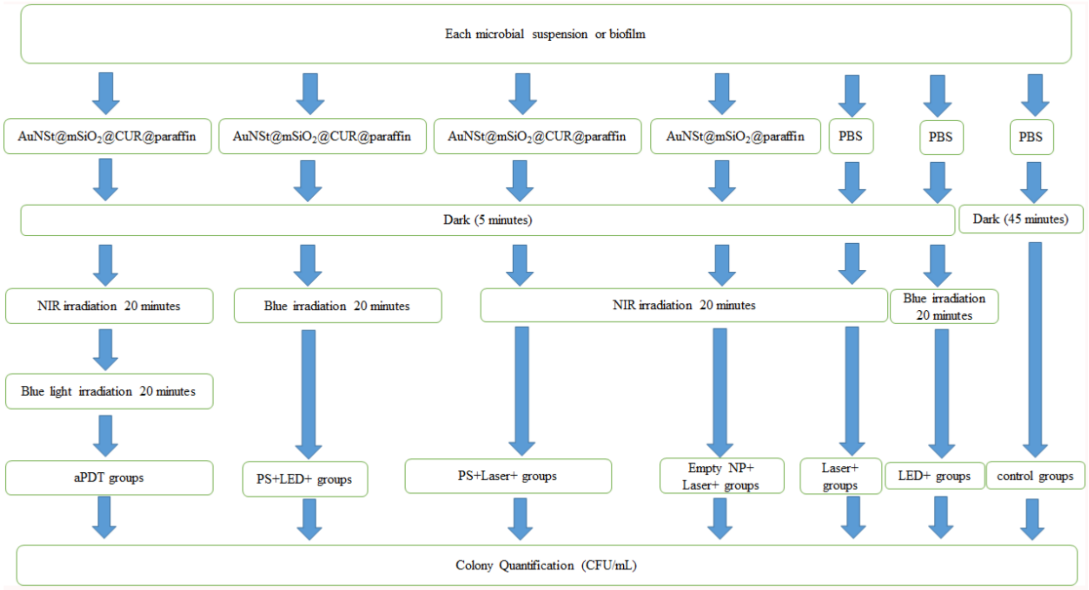

Figure S1. Experimental conditions for aPDT against bacterial planktonic cultures and biofilms.

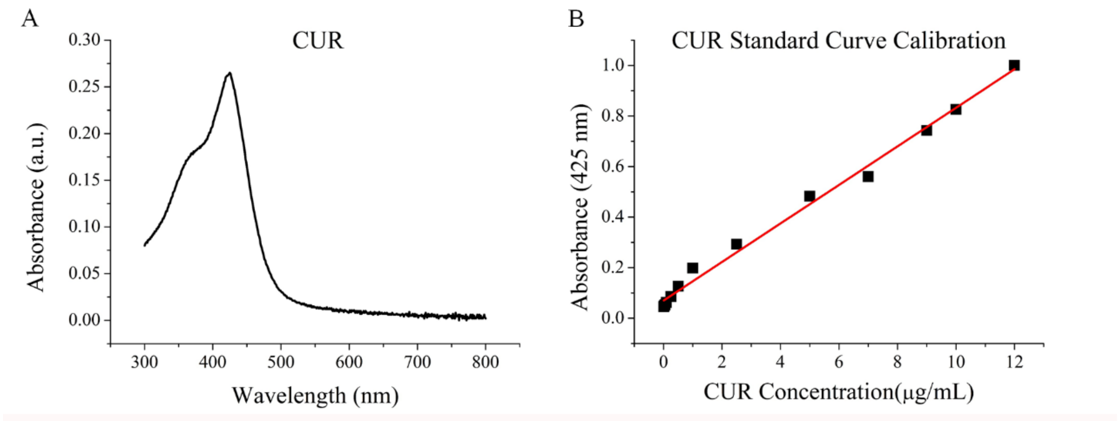

Figure S2. Curcumin absorption spectrum (a) and Curcumin standard curve calibration (b).
